# Future of biobanks – bigger, longer, and more dimensional

**DOI:** 10.3325/cmj.2013.54.496

**Published:** 2013-10

**Authors:** Ozren Polašek

**Affiliations:** Centre for Global Health, Medical School, University of Split, Split, Croatia

Collections and repositories of biological samples that are used for research and medicinal purposes are referred to as biobanks. Biobanks focused on genetic epidemiology are currently dominating the field of biomedical research. This is clearly seen in the research priorities across Europe, where numerous biobanks have been developed and supported (1,2). Even the most superficial overview of these biobanks suggests a large variety of approaches in terms of their size, target population, sampling scheme, phenotyping extent, or biological samples that are being collected. Majority of biobanks are sampling apparently healthy subjects, who may be originating either from general or special populations. Special populations like isolated island populations are highly interesting research targets, due to their greater chances for successful gene mapping (3), arising from the isolated populations’ genetic structure (4).

## Research potential

Research potential is one of the main reasons why biobanks have received substantial supports from the financing bodies. Since individual biobanks may have small potential to contribute to the top-level research due to limited sample size, joining of biobanks presents an ideal approach to overcome this problem. One of the early examples of such activities that was recognized and used in research was an FP6 project EUROSPAN, a consortium of five biobanks of isolated populations across Europe (*http://www.eurospan.org*). The project offered an initial boost to isolated populations biobanks development and yielded a substantial research output (5-7). Similar initiatives were formed elsewhere, producing consortia of both regional coverage such as BBMRI, or even global coverage such as the CHARGE, GIANT, or MAGIC. Such consortia often produce top-level research results, based on hundreds of thousands of samples analyzed in a meta-analytic approach (8-11). At the same time, some biobanks were conceived and managed to provide huge sample sizes, including UK Biobank or Chinese Kadoorie Biobank, both with over half a million of samples (12). Either by developing new biobanks or joining the existing ones, one of the first properties of the future biobanks is apparently the provision of large enough sample sizes and growth of the existing biobanking potential.

## Population participation

One of the crucial, yet often overlooked issues in biobanking is the potential benefit of the sampled population. Examples of “helicopter genetics,” where researchers did not provide any benefit for the target population are now behind us. Recent studies have identified the reasons for individuals’ participation in biobanking (13,14), but such reasons could be locally specific, thus requiring a detailed case-by-case analysis. The biobanks of tomorrow (and today) will have to incorporate population perception and benefit high on their priority list. However, studies on this issue are very scarce (15) and this is one of the research areas that should be developed further in the near future.

The Croatian Biobank – The 10 001 Dalmatians is an example of an isolated population biobank, with a focus on genetic basis of complex diseases (4,16). Although the target sample size has not been reached yet, the biobank currently contains biological samples of about 4500 subjects from six Croatian islands (Vis, Korčula, Mljet, Lastovo, Rab, Susak) and the coastal city of Split. This resource currently collects over 800 phenotypic measurements, including blood, sera, and urine samples coupled with dried blood spots (17), anthropometrical and clinical measurements, a wide set of biochemical measurements including over 240 lipidomic fractions, over 140 N-glycans, extensive survey information and cognition-based traits. Furthermore, two new lines of measurements have been recently initiated, “sensomics” and “anthropomics.” The first includes hearing, smell, taste, and pain thresholds, coupled with a large number of perception and cognition measurements obtained from the complex reactiometer Drenovac (CRD) apparatus (18,19). The second, “anthropomics,” includes detailed imaging techniques, with cone-beam tomography of the teeth and a 3D scan of the face, while 3D scan of the entire body surface is planned in the future. In addition to these developments, a recent collaboration with the Croatian National Institute of Public Health has resulted in the linking of national mortality data with the biobank data, thus offering broader and more versatile research information on causes of death and enabling prospective nature of the data collection process. In the future, the biobank will also try to link the data for every participant with the data from the health system, thus enabling an up-to-date source of relevant medical information and the possibility to provide initial steps of developing integrated framework of personalized medicine (20-22). In addition, subsets of the initial sample have already been followed-up and this practice will continue in the future. Therefore, two additional properties of the future biobanks emerge – conversion into cohort-based or follow-up study design and increasing dimensionality of the phenotypes that are being measured.

Further steps to increase the research potential of the 10 001 Dalmatians will be based on three lines of research: a) systems biology, which aims to understand the total complexity of biological processes in the human body (23,24), b) analysis of pleitropic effects and better understanding of gene networks, and c) possibility to do a detailed phenome-wide association studies, with the aim of identifying associations of a large number of phenotypes with genes, in an inverse approach to the commonly used genome-wide association studies. An additional focus of the 10 001 Dalmatians project will be complementation of the existing population genetics data (25-28) in order to create a systematic and comprehensive overview of the genetic structure of the modern Croatians.

## Maternal and child biobanks

Majority of biobanks in the high income countries are focusing on adult donors. Furthermore, such biobanks are often prone to selection bias, as they tend to sample apparently healthy subjects and omit the whole spectrum of early-onset diseases and early cases of mortality. A possible step forward could be development of biobanks focused on maternal and child health in low- and middle-income countries (29-31), ensuring useful policy information and ultimately providing a basis for health care improvements in populations that need them the most (32-35).

## Genes vs environment

Although a list of specific and interesting biobanks that were recently developed could indeed be lengthy, two recent examples require a mention in the context of genes and environment interactions. The first one is related to the project DENAMIC (Developmental Neurotoxicity Assessment of Mixtures in Children; *http://www.denamic-project.eu*), which investigates neurotoxic effects of low-concentration mixtures of pesticides and a number of common environmental pollutants in children. The focus of the project is to investigate sub-clinical effects on learning, cognitive skills, and developmental disorders in children, in the domain of attention-deficit hyperactivity disorder, autism, and anxiety disorders. The second example is PREPARE (Platform foR European Preparedness Against (Re-)emerging Epidemics; *http://www.prepare-europe.eu/*), which includes the development of a biobank that will collect samples of humans who have developed severe cases of respiratory infections, thus enabling better understanding of the diseases pathology and host susceptibility.

It would therefore seem that over time biobanks have gone beyond simple cross-sectional collections of human samples used to investigate the genetic basis of disease toward broader set of health risks, including environmental exposures like chemical and biological risk factors.

## Ethics and governance

Ethical background for biobanks creation and utilization remains to be one of the topics that are insufficiently discussed (36). Various issues have been recognized, including problems with the sample ownership and biobank governance (37-39), some of which have prompted a completely new line of research on bio-objects (40-44). Current situation surely requires systematic attempts to develop a common framework, enabling better and wider use of biobanks. Ultimately, biobanks will create a climate which will give rise to the true development of personalized medicine (45-47), which will become especially interesting in the domain of aging and complex diseases genetics (48).

## Future directions

Several trends in the biobanks development are now becoming visible. A tendency of converting biobanks into the cohort-based studies or at least follow-up studies is becoming increasingly more prevalent. One such initiative is BBMRI-LPC (Biobanking and Biomolecular Research Infrastructure – Large Prospective Cohorts; *http://www.bbmri-lpc.org/*), which aims to strengthen, harmonize, and link together longitudinal biobanks in Europe. The clear advantage of this approach resides in the time-related exposures and the possibility to predict end-outcomes based on the individual genetic make-up. This example also shows an inherent need to harmonize and enable cooperation of biobanks, thus producing competitive joint resources for biomedical research.

In conclusion, it seems that biobanks of the future will have to provide participative conditions and become more oriented toward the population they are sampling. In turn, target populations should have indirect (overall research advantages) and direct benefits from the biobanks, in the form of better understanding of health determinants in a specific population (49). The best possible scenario, especially in the small and isolated populations, is development of targeted preventive activities, based on the results originating from the biobanks development. This process should be closely aligned with health care system activities, which should also provide existing morbidity and mortality data for better utilization of biobanks. All these premises suggest that biobanks should become and remain adaptive and be constantly developing new approaches and new ideas. Despite numerous uncertainties, it seems very likely that the biobanks of the future will become bigger in size, longer in duration, and that the phenotyping focus will include increasing dimensionality, in the form of more complex, repeated, or life-long measurements, as well as the possibility to investigate environmental influences on health and disease.
